# Acute Effects of a Multi-Ingredient Preworkout Supplement on Peak Torque and Muscle Excitation During an Isokinetic Fatigue Protocol

**DOI:** 10.3390/sports13110404

**Published:** 2025-11-11

**Authors:** Benjamin R. Connors, Clayton L. Camic, Andrew R. Jagim, Christopher M. Hill, Emerson Sebastião, Peter J. Chomentowski, Rachel A. Kowal, Matteo F. de Leon

**Affiliations:** 1 Department of Kinesiology and Physical Education, Northern Illinois University, DeKalb, IL 60115, USA; ben.connors.1996@gmail.com (B.R.C.); pchomentowski@niu.edu (P.J.C.); rkowal1@niu.edu (R.A.K.); z1927462@students.niu.edu (M.F.d.L.); 2 Sports Medicine, Mayo Clinic Health System, Onalaska, WI 54650, USA; jagim.andrew@mayo.edu; 3 School of Kinesiology, Louisiana State University, Baton Rouge, LA 70803, USA; chrishill@lsu.edu; 4 Department of Health and Kinesiology, University of Illinois Urbana-Champaign, Urbana, IL 61820, USA; esebast2@illinois.edu

**Keywords:** isometric, concentric, eccentric, leg extensors, quadriceps, electromyography

## Abstract

The primary purpose of this study was to examine the acute effects of a multi-ingredient preworkout supplement (MIPS) on isometric, concentric, and eccentric peak torque and electromyographic (EMG) responses of the leg extensors during a fatiguing isokinetic protocol. Thirteen male subjects (mean age ± SD = 22.9 ± 2.2 years) were assigned in crossover fashion to ingest an MIPS or placebo before an isokinetic protocol that consisted of 30 maximal, concentric and eccentric muscle actions with EMG signals recorded from the vastus lateralis, rectus femoris, and vastus medialis muscles. Immediately before (PRE) and after (POST) the isokinetic fatigue protocol, subjects were assessed for isometric peak torque. The MIPS condition resulted in greater isometric (205 ± 48 vs. 185 ± 44 N·m, *p* = 0.04) and concentric (121 ± 34 vs. 103 ± 27 N·m, *p* = 0.015) torque values versus placebo (collapsed across time). For eccentric peak torque as well as EMG amplitude and frequency values, there were no significant (*p* > 0.05) interactions or main effects for each condition. These findings indicated that acute ingestion of the MIPS enhanced isometric and concentric peak torque of the leg extensors, which was not explained by changes in the EMG signal.

## 1. Introduction

In the past several years, the use of multi-ingredient pre-workout supplements (MIPS) has become extremely popularized, and their ergogenic potential has become well-established for various sports and within the general population by both competitive and recreational athletes [1,2,3,4,5,6]. Some of the more prevalent ingredients in MIPS include caffeine, β-alanine, L-citrulline, L-arginine, and creatine, with many of these ingredients and others often combined into proprietary blends [7]. Although recent findings indicate synergistic effects among these ingredients may be present [8], it has been suggested that caffeine is the predominant factor responsible for the acute ergogenic effects associated with MIPS [2,9]. In fact, the recommended timing for ingestion of most MIPS is likely based on the stimulating effects of caffeine, which typically peaks in the bloodstream 30–60 min post-ingestion [10,11]. As a nutritional supplement, caffeine has been shown to be beneficial for maintaining maximal strength and endurance as well as delaying the onset of fatigue and improving time-trial performance along with other forms of high-intensity exercise [10,11]. These effects have been attributed to improved motor unit firing rates and calcium release from the sarcoplasmic reticulum, subsequently enhancing muscle contraction force [4,12]. β-alanine is another ingredient included in most MIPS [7] and has been identified as the rate-limiting precursor to carnosine, an intracellular muscle buffer [13]. Supplementation with β-alanine has been shown to increase intramuscular levels of carnosine, thereby attenuating metabolic acidosis and contributing to improvements in exercise capacity during high-intensity effort [13]. As precursors to the potent vasodilator, nitric oxide, both L-citrulline and L-arginine supplements have been demonstrated to enhance blood flow to active muscles, subsequently delaying the onset of muscular fatigue [14,15,16]. Based on these collective physiological mechanisms, an MIPS containing these notable ergogenic ingredients (caffeine, β-alanine, L-citrulline, L-arginine) may provide neuromuscular benefits for sustaining force or power output during various forms of vigorous activity.

The majority of previous investigations examining the acute effects of MIPS on performance have focused on isotonic variables of upper and lower body muscular strength [i.e., bench press, leg press, and squat one-repetition maximum (1-RM), maximum voluntary contractions (MVC)], endurance (i.e., repetitions to failure at %1-RM), and power (vertical jump, Wingate Anaerobic testing, and critical power) [1,2,3,4,5,6,9,12,17,18]. Despite conflicting evidence for maximal force and power production [4,6,19,20], the findings of several studies [1,3,4,12,17,18,21,22,23] have largely indicated that acute MIPS supplementation can enhance overall force retention and muscular endurance during prolonged or intermittent bouts of high-intensity activity. Currently, however, the exact underlying mechanisms responsible for these benefits on neuromuscular function are poorly understood.

Although isotonic resistance training offers ecological validity and practical applicability, its outcomes can be substantially influenced by skill level and movement technique. Isokinetic dynamometers coupled with surface electromyography (EMG) recordings are two frequently utilized instruments for the non-invasive assessment of neuromuscular function and fatigue during static and dynamic muscle actions [24,25,26,27]. The utility of isokinetic testing involves the ability to measure isometric, concentric, and eccentric torque levels across an entire range of motion at controlled velocities with minimal skill involvement required. Results from isokinetic testing can provide torque production at different joint angles, while identifying muscle imbalances, tracking the recovery process from injury or surgery, and measuring the rate of force development [28,29]. Thus, as both a training and assessment tool, isokinetic testing is common in clinical and performance settings.

Surface electromyography (EMG) is a technique that involves recording and quantifying the action potentials associated with contracting skeletal muscle fibers [30]. The amplitude contents of the EMG signal reflect the level of muscle excitation [31], whereas the frequency contents provide information related to the muscle fiber conduction velocity [32]. To our knowledge, only one study [5] has directly examined EMG responses associated with acute MIPS administration. Specifically, Negro et al. [5] reported that acute MIPS (creatine, arginine, β-alanine, glutamine, taurine) ingestion improved EMG-based indicators of fatigue (i.e., conduction velocity, fractal dimension) during sustained isometric contractions following a resistance exercise protocol designed to elicit fatigue. The authors [5] proposed that these acute benefits of their MIPS product may be attributable to improved: (1) peripheral components of performance fatiguability, (2) buffering capacity of the muscle from β-alanine, and (3) regulation of mechanisms associated with exercise-induced fatigue from arginine, glutamine, and taurine. Based on these findings [5], valuable insight can be gained into the underlying neuromuscular factors associated with acute MIPS ingestion by examining muscle function and fatigue through isokinetic and EMG assessments.

Despite the popularity of MIPS and well-documented benefits on isotonic muscular performance, there is limited research on the effects of these products on isokinetic function and neuromuscular excitation. In addition, the influence of MIPS on different types of muscle actions (isometric, concentric, and eccentric) under both rested and fatigued conditions is poorly understood. Due to differences in metabolic cost, neural drive, and mechanical efficiency among these contraction types [33] that are fundamental to sports performance and rehabilitative exercise, understanding whether MIPS ingestion exerts muscle action-specific effects remain an important and unexplored question. Thus, the primary purpose of the present study was to examine the acute effects of an MIPS product on isometric, concentric, and eccentric peak torque production of the leg extensors during a fatiguing isokinetic protocol. In addition, we investigated the effects of the MIPS supplement on EMG amplitude and median power frequency (MDF) responses from the vastus lateralis, rectus femoris, and vastus medialis muscles.

## 2. Materials and Methods

### 2.1. Study Design

Prior to data collection, a brief pilot trial (*n* = 3) was performed to refine familiarization and testing procedures. A randomized, double-blind, placebo-controlled, within-subjects experimental design was used for the present study. Each subject visited the laboratory for three sessions, with a week between each session. The first visit required subjects to practice a series of submaximal and maximal isometric, concentric, and eccentric muscle actions of the leg extensors on a calibrated isokinetic dynamometer to familiarize the subjects with the testing procedures. For the second visit, each subject was randomly assigned to ingest one serving of the MIPS or one serving of the placebo 30 min before completing an isokinetic fatigue protocol with EMG signals recorded from superficial muscles of the quadriceps. For the third visit, subjects ingested the other substance not administered during the second visit and repeated the same testing protocol and procedures. Two-day food logs were recorded prior to every visit with the MyFitnessPal app (MyFitnessPal, Inc., Baltimore, MD, USA).

### 2.2. Subjects

**Figure 1:**
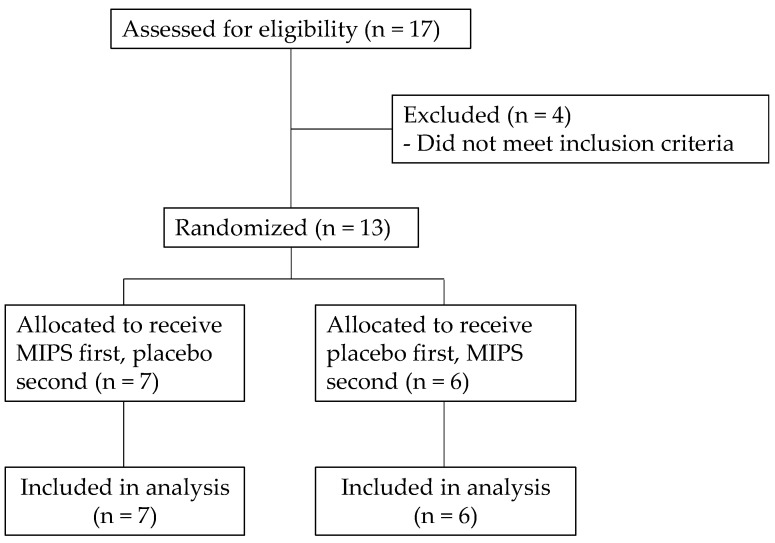
Consolidated Standards of Reporting Trials (CONSORT) flow diagram.

Thirteen recreationally trained male subjects (mean age ± SD = 22.9 ± 2.2 years; body mass = 84.6 ± 8.6 kg; resistance training = 5.0 ± 3.4 hr·wk^−1^; resistance training: 5.0 ± 3.4 h per week; aerobic training: 1.7 ± 1.1 h per week) volunteered for this investigation (Figure 1). Subject recruitment was completed through flyers posted at Northern Illinois University seeking resistance-trained males for a supplementation study on muscular strength. To achieve a power (1-β) of 0.8 with an effect size of 0.3 and alpha of 0.05 for a within-subjects and crossover experimental design, a sample size of at least 12 was calculated (G*Power 3.1, Heinrich-Heine-Universität Düsseldorf, Düsseldorf, Germany). Eligibility requirements included no history of cardiovascular, musculoskeletal, or metabolic diseases; no use of medications or supplements in the last 30 days that would influence study outcomes; and average caffeine intake of less than one caffeinated drink per day. The subjects were instructed to refrain from exercise for 48 h as well as eating or drinking anything other than water for three hours before laboratory visits 2 and 3 [17]. In addition, the subjects were asked to completely abstain from caffeine use for at least two weeks before starting the investigation [8]. The study was conducted according to the guidelines of the Declaration of Helsinki, approved by the Institutional Review Board of Northern Illinois University (#HS22-0139, 15 November 2021), and registered on clinicaltrials.gov (NCT07217210). Prior to all study procedures, subjects signed an informed consent and filled out a health history questionnaire.

### 2.3. Procedures

#### 2.3.1. Visit 1: Familiarization

The first visit involved subject familiarization with instructions for the study (e.g., exercise restrictions, using MyFitnessPal (MyFitnessPal, Inc., Baltimore, MD, USA) for tracking food intake, etc.) and testing procedures (isokinetic dynamometer exercises, EMG electrode placement and procedures, etc.). Specifically, subjects practiced submaximal and maximal isometric, concentric, and eccentric muscle actions of the leg extensors on an isokinetic dynamometer (Humac Norm, Computer Sports Medicine, Inc., Stoughton, MA, USA) [24,25,26,27]. The joint angle of the knee was maintained at 120° for all isometric muscle actions [25]. The isokinetic velocity for concentric and eccentric muscle actions was constant at 60°·s^−1^.

#### 2.3.2. Visits 2 and 3: Fatigue Protocol Tests with Supplementation

Supplementation Protocol. During visits 2 and 3, subjects were provided either one serving of the MIPS (Beyond Raw Lit, General Nutrition Company, Pittsburgh, PA, USA) or placebo (Great Value artificially flavored, noncaloric drink mix; Walmart Stores, Inc., Bentonville, AR, USA) using randomized and double-blind administration procedures. In addition, the placebo was matched for flavor with the MIPS. Based on the recommendations of the MIPS manufacturer, substances were ingested with a glass of water 30 min before the testing procedures. Ingredients of the MIPS included: (1) CarnoSyn Beta-Alanine (3.2 g), (2) Caffeine Anhydrous (250 mg), (3) L-citrulline (3.0 g), (4) L-arginine (1.5 g), (5) elevATP (Ancient Pear and Apple Extract) (150 mg), and (6) Neurofactor (Coffea arabica) (fruit extract) (100 mg).

Fatigue Protocol. All subjects completed a warm-up of 10, 3-s isometric muscle actions as well as 10 concentric and 10 eccentric muscle actions at 60°·s^−1^ corresponding to approximately 50–75% of their subjective maximum effort [24,25,26,27]. Following the warm-up and two minutes of rest, the subjects performed two 3-s isometric MVCs at a joint angle of 120° between the thigh and leg. Each isometric MVC was separated by 5 s of rest. The average of these first two MVC values was used as the representative baseline (PRE) MVC score. After another two minutes of rest, the subjects completed a total of 30 consecutive, maximal concentric and eccentric muscle actions at 60°·s^−1^. Specifically, the subject started with their leg at 90° flexion and performed a maximal concentric muscle action to full extension (180°). Once full extension was attained, a maximal eccentric muscle action was performed from full extension (180°) back to 90° flexion. Immediately following the maximal eccentric muscle action, this process of repeating maximal concentric, followed by maximal eccentric muscle actions, continued until 30 concentric and eccentric repetitions had been completed. The representative PRE and POST peak torque values for the concentric and eccentric muscle actions were calculated as an average of the first three muscle actions and an average of the last three muscle actions, respectively. Immediately after the fatigue protocol, two additional 3-s isometric MVCs were completed with 5 s rest between each MVC. The average of these last two MVC values was used as the representative POST MVC score. The range of motion was standardized to 90° to 180° at the knee for all subjects [27]. During each maximal muscle action, the subjects were provided with verbal encouragement to produce as much torque as possible.

EMG Electrode Placement and Signal Processing. Visits 2 and 3 utilized SENIAM recommendations [34] to locate the EMG electrode (Tringo Wireless EMG, Delsys Inc., Natick, MA, USA) placements for the vastus medialis, rectus femoris, and vastus lateralis. The wireless electrodes were adhered to the skin with double-sided stickers after the skin was shaved, lightly abraded with gauze, and cleaned with isopropyl alcohol. Tringo Wireless EMG system (Delsys Inc., Natick, MA, USA) was used to record the EMG signals using an amplification of gain ×1000 and sampling frequency of 2000 Hz. EMG signals were recorded through the entire test and stored in a personal computer (Dell Latitude 5480, Round Rock, TX, USA) for further processing [25,26]. MATLAB software (version 9.10 (2021A), Mathworks, Natick, MA, USA) was used for all signal analyses using custom programs. One-second epochs were selected for each muscle action and used to calculate EMG amplitude (μVrms) and median power frequency values (MDF, Hz) [25,26]. These EMG amplitude and MDF values of each muscle action were then normalized to their representative PRE isometric maximum voluntary contraction value.

### 2.4. Statistical Analyses

All statistical analyses were completed using SPSS software program (version 29, IBM Corp., Armonk, New York, USA). All data are presented as mean ± SD. Two-way repeated-measures analyses of variance (ANOVAs) were used to determine significant mean differences in peak torque, normalized EMG amplitude, and normalized EMG MDF among conditions (MIPS, placebo) and time (PRE, POST). When appropriate, follow-ups included paired-sample *t*-tests. All daily energy intake (kcals) and macronutrient data (grams of carbohydrate, fat, and protein) were analyzed with paired-sample *t*-tests between conditions (MIPS vs. placebo). An alpha of <0.05 was considered statistically significant for all analyses.

## 3. Results

### 3.1. Peak Torque Production

#### 3.1.1. Isometric Peak Torque

**Figure 2:**
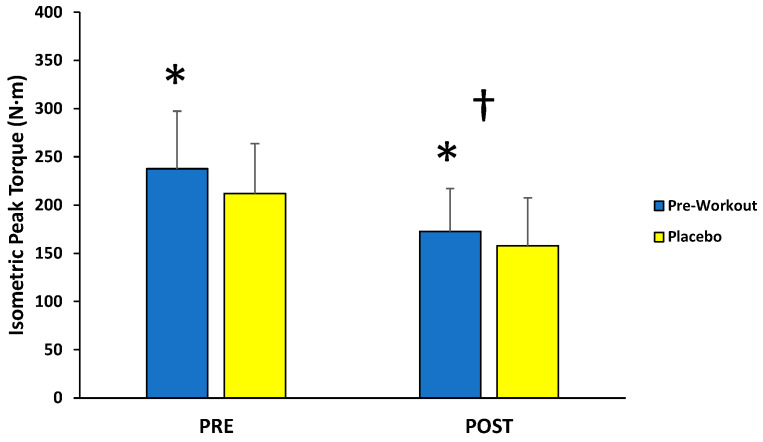
Peak torque values (mean ± SD) for maximal isometric muscle actions of the leg extensors performed before (PRE) and after (POST) the isokinetic fatigue protocol. * Main effect for condition (MIPS > Placebo). † Main effect for time (PRE > POST).

For isometric MVCs, there was no significant interaction (*F*(1,12) = 1.095; *p* = 0.316; partial η^2^ = 0.084) for condition (MIPS, placebo) across time (PRE, POST), but there were main effects for condition (*F*(1,12) = 5.066; *p* = 0.044; partial η^2^ = 0.297) and time (*F*(1,12) = 26.477; *p* < 0.001; partial η^2^ = 0.688) (Figure 2). Follow-up paired-sample *t*-tests indicated the MIPS condition (205 ± 48 N·m) resulted in significantly greater torque compared to the placebo (185 ± 44 N·m) collapsed across time, whereas the PRE torque values (225 ± 53 N·m) were significantly greater than POST torque values (165 ± 42 N·m) collapsed across condition.

#### 3.1.2. Concentric Peak Torque

**Figure 3:**
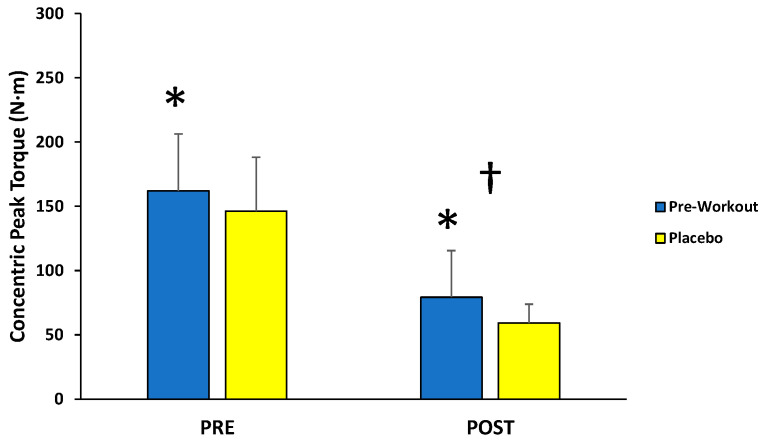
Peak torque values (mean ± SD) for maximal concentric muscle actions of the leg extensors performed before (PRE) and after (POST) the isokinetic fatigue protocol. * Main effect for condition (MIPS > Placebo). † Main effect for time (PRE > POST).

For concentric peak torque production, there was no significant interaction (*F*(1,12) = 0.099; *p* = 0.759; partial η^2^ = 0.008) for condition (MIPS, placebo) across time (first 3, last 3), but there were main effects for condition (*F*(1,12) = 8.040; *p* = 0.015; partial η^2^ = 0.401) and time (*F*(1,12) = 95.157; *p* < 0.001; partial η^2^ = 0.888) (Figure 3). Follow-up paired-sample *t*-tests indicated the MIPS condition (121 ± 34 N·m) resulted in significantly greater torque compared to the placebo (103 ± 27 N·m) collapsed across time, whereas the PRE torque values (154 ± 40 N·m) were significantly greater than POST torque values (69 ± 21 N·m) collapsed across condition.

#### 3.1.3. Eccentric Peak Torque

**Figure 4:**
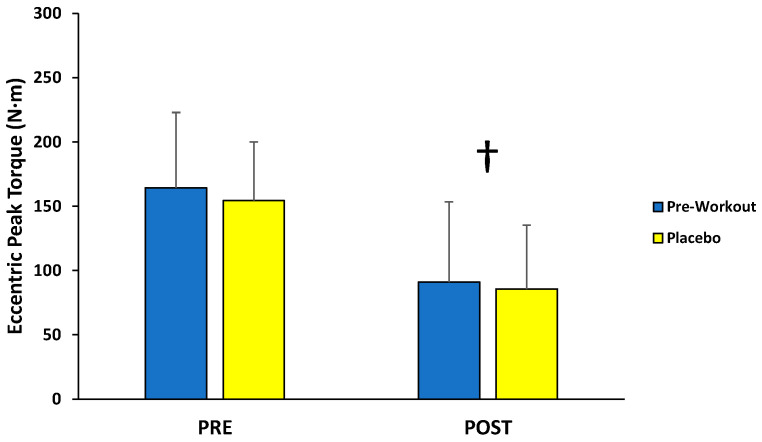
Peak torque values (mean ± SD) for maximal eccentric muscle actions of the leg extensors performed before (PRE) and after (POST) the isokinetic fatigue protocol. † Main effect for time (PRE > POST).

For eccentric peak torque production, there was no significant interaction (*F*(1,12) = 0.110; *p* = 0.746; partial η^2^ = 0.009) or main effect for condition (*F*(1,12) = 1.198; *p* = 0.295; partial η^2^ = 0.091), but there was a main effect for time (*F*(1,12) = 40.160; *p* < 0.001; partial η^2^ = 0.770) (Figure 4). The follow-up paired-sample *t*-test indicated the PRE torque values (159 ± 49 N·m) were significantly greater than the POST torque values (88 ± 54 N·m) collapsed across condition.

### 3.2. EMG Amplitude

#### 3.2.1. Isometric Muscle Actions

There were no significant (*p* > 0.05) interactions or main effects for condition, but there main effects for time for the vastus medialis (*F*(1,11) = 5.719; *p* = 0.036; partial η^2^ = 0.342), rectus femoris (*F*(1,11) = 7.579; *p* = 0.019; partial η^2^ = 0.408), and vastus lateralis (*F*(1,11) = 8.414; *p* = 0.014; partial η^2^ = 0.433) (Table 1). Follow-up paired-sample *t*-tests indicated the mean POST EMG amplitude values for the vastus medialis, rectus femoris, and vastus lateralis (145 ± 65, 149 ± 62, and 143 ± 52%, respectively) were significantly greater than the PRE (100 ± 0, 100 ± 0, 100 ± 0%, respectively) (collapsed across conditions).

#### 3.2.2. Concentric Muscle Actions

For the vastus medialis, rectus femoris, and vastus lateralis, there were no significant (*p* > 0.05) interactions or main effects for condition or time (Table 1).

#### 3.2.3. Eccentric Muscle Actions

For the vastus medialis, there was a significant interaction (*F*(1,11) = 6.429; *p* = 0.028; partial η^2^ = 0.369) (Table 1). Follow-up paired-sample *t*-tests indicated no significant mean differences in PRE EMG amplitude values (MIPS: 148 ± 112% vs. placebo: 104 ± 32%, *p* = 0.052) or POST EMG amplitude values (MIPS: 156 ± 134% vs. placebo: 145 ± 83%, *p* = 0.673) between conditions. For the rectus femoris and vastus lateralis, there were no significant (*p* > 0.05) interactions or main effects for condition or time.

### 3.3. Median Power Frequency

#### 3.3.1. Isometric Muscle Actions

For the vastus medialis, rectus femoris, and vastus lateralis, there were no significant (*p* > 0.05) interactions or main effects for condition or time (Table 2).

#### 3.3.2. Concentric Muscle Actions

For the vastus medialis and rectus femoris, there were no significant (*p* > 0.05) interactions or main effects for condition, but there were main effects for time (Table 2). The follow-up paired-sample *t*-tests indicated the mean PRE EMG MDF values for the vastus medialis and rectus femoris (105 ± 13 and 105 ± 21%, respectively) were significantly greater than the POST (98 ± 15 and 89 ± 24%) (collapsed across conditions). For the vastus lateralis, there was no significant (*p* > 0.05) interaction or main effects for condition or time.

#### 3.3.3. Eccentric Muscle Actions

For the vastus medialis, rectus femoris, and vastus lateralis, there were no significant (*p* > 0.05) interactions or main effects for condition or time (Table 2).

### 3.4. Food Log Data

There were no significant differences between conditions for daily energy intake (MIPS: 2123 ± 476 vs. placebo: 1996 ± 330 kcals, *p* = 0.083), carbohydrate (MIPS: 193 ± 54 vs. placebo: 179 ± 55 g, *p* = 0.113), fat (MIPS: 91 ± 30 vs. placebo: 84 ± 21 g, *p* = 0.202), or protein (MIPS: 150 ± 51 vs. placebo: 148 ± 37 g, *p* = 0.860) ingestion.

## 4. Discussion

This study examined the acute effects of MIPS ingestion on muscle action-specific peak torque and EMG responses of the leg extensors during a fatiguing isokinetic protocol. As demonstrated by the main effects for time, the isokinetic protocol resulted in fatigue-induced decreases in peak torque from PRE to POST for isometric (−27%), concentric (−55%), and eccentric (−45%) muscle actions. The main findings of the present study indicated that an acute dose of the MIPS significantly improved isometric (+11%) and concentric (+17%) peak torque production of the leg extensors before (PRE) and after (POST) the fatigue protocol compared to placebo, but not eccentric peak torque. In addition, these beneficial effects on isometric and concentric peak torque were not explained by EMG amplitude or MDF responses of the vastus lateralis, rectus femoris, or vastus medialis, which demonstrated no significant differences between the MIPS and placebo conditions.

To our knowledge, this is the first study to examine the acute effects of an MIPS product on isokinetic fatigue-induced changes in isometric peak torque. Specifically, the MIPS condition (205 ± 48 N·m) resulted in greater isometric torque compared to the placebo (185 ± 44 N·m) (collapsed across time). Bioactive compounds in the current MIPS product included caffeine (250 mg or 3 mg·kg^−1^), β-alanine (3.2 g), citrulline (3.0 g), and arginine (1.5 g). As a mild central nervous system stimulant and primary active ingredient in most MIPS products [4,7], caffeine provides potential ergogenic effects during exercise including: (1) enhanced endurance performance by blocking adenosine receptors resulting in reduced perception of effort and pain [11,35,36], and (2) greater muscular strength and power by increasing motor unit activation through firing rates and promoting greater release of calcium from the sarcoplasmic reticulum leading to more crossbridge formation [4,11,37]. Although provided at approximately half the recommended ergogenic dose when administered in isolation [7], the inclusion of both citrulline (3.0 g) and arginine (1.5 g) in the present MIPS may have improved blood flow [38,39], thereby attenuating fatigue-induced decreases in peak torque. As previously suggested [5,40], it is also possible that β-alanine can function on an acute basis by increasing cytosolic calcium levels and serving as an intracellular buffer during high-intensity exercise. Collectively, the primary ingredients (caffeine, β-alanine, citrulline, and arginine) in the current MIPS provide numerous physiological mechanisms that potentially contributed to the improved isometric peak torque values.

Stratton et al. [18] reported that both a caffeinated MIPS (caffeine, 350 mg; L-citrulline DL-malate 2:1, 8 g; β-alanine 3.6 g; betaine anhydrous, 2.5 g; L-theanine, 350 mg; alpha-glyceryl phosphorylcholine, 300 mg) and an identical noncaffeinated product resulted in greater squat isometric peak force values following acute ingestion compared to placebo. These findings [18] strongly suggested caffeine may not be solely responsible for the ergogenic effect on isometric force. In contrast, Beyer et al. [1] demonstrated that acute MIPS supplementation (L-citrulline, 8 g; creatine monohydrate, 5 g; taurine, 3 g; β-alanine, 2.5 g; betaine anhydrous, 2.5 g; L-tyrosine, 2 g; alpha-glyceryl phosphorylcholine, 300 mg; caffeine, 300 mg; L-theanine, 150 mg) had no effect on isometric mid-thigh pull in a placebo-controlled, crossover study. Negro et al. [5] examined the acute effects of an MIPS product containing creatine (3 g), arginine (2 g), β-alanine (0.8 g), glutamine (1 g), taurine (1 g) on isometric MVCs and 60% MVC until exhaustion in the biceps brachii with EMG measurements before and after completing a resistance exercise protocol designed to induce fatigue. The authors [5] demonstrated no significant PRE to POST changes in MVC or motor unit synchronization, but improved time to exhaustion at 60% MVC and enhanced fiber conduction velocity of the motor unit action potentials for the MIPS condition compared to placebo. Thus, the beneficial effects of MIPS on a sustained isometric contraction in the study of Negro et al. [5] were partially explained by neuromuscular factors of the EMG signal. In the present investigation, however, the greater isometric strength in the MIPS condition could not be attributed to changes in EMG amplitude or MDF, which reflect levels of muscle excitation and motor unit conduction velocity, respectively [31,32]. Due to diverse MIPS ingredient formulations (specific ingredients, dosages, proprietary blends) utilized among different studies, making direct comparisons of results remains challenging and is commonly addressed in the literature [1,2,3,5,6,9,18]. Recently, however, Montalvo-Alonso et al. [41] demonstrated that acute caffeine intake (3 mg·kg^−1^) enhanced muscular strength and power at 75–90% 1-RM during the back squat with no subsequent changes in EMG activity in the vastus lateralis and rectus femoris muscles. Kalmar and Cafarelli [42] also reported caffeine (6 mg·kg^−1^) increased isometric MVC of the knee extensors despite no improvement in H-reflex amplitude, EMG amplitude, or motor unit firing rates. In contrast, Behrens et al. [43] found acute caffeine supplementation at 8 mg·kg^−1^ increased isometric MVC of the leg extensors in combination with enhanced voluntary activation and normalized muscle activity. The authors [43] proposed that caffeine ingestion leads to an augmented neural drive at the supraspinal level, thereby improving isometric MVC strength. Thus, the findings of the present study and those of others [41,42,43] suggested that small to large doses of caffeine (3–8 mg·kg^−1^) increase isometric peak torque production of the leg extensors, but these ergogenic effects may not be reflected in EMG responses below 8 mg·kg^−1^.

The improved isometric and concentric peak torque production associated with MIPS ingestion without subsequent changes in EMG amplitude or MDF suggested the ergogenic effect may be the result of enhanced central mechanisms or excitation-contraction coupling rather than greater peripheral neural drive. For example, the findings of Walton et al. [44] demonstrated that caffeine may improve peak torque through enhanced supraspinal mechanisms that augment voluntary activation in the absence of associated increases in EMG amplitude at the local surface. Furthermore, it has been shown [45] that caffeine ingestion can delay the onset of isometric fatigue without altering motor unit firing rates. Thus, the peripheral effects of caffeine (increased release of calcium from the sarcoplasmic reticulum) may provide greater torque output relative to neural input [45]. It is also possible that the small yet ergogenic dose of caffeine (250 mg or approximately 3 mg·kg^−1^ of body mass) in the MIPS was below the threshold for observable changes in muscle excitation. Although the 30-min ingestion timing prior to exercise was utilized for the present MIPS due to manufacturer recommendations, it is plausible that caffeine may have exerted a greater neuromuscular effect 45–60 min after ingestion [44].

In theory, the physiological mechanisms associated with arginine, citrulline, and β-alanine could enhance force retention during intense exercise, but these effects are likely contingent upon sufficient dosing or chronic administration. Both arginine and citrulline are known to enhance nitric oxide synthesis, thereby promoting blood flow and delaying the onset of neuromuscular fatigue [14,15,16]. However, the amounts of arginine (1.5 g) and citrulline (3.0 g) in the current MIPS are half of the recommended ergogenic acute doses of ≥3–6 g and ≥6 g, respectively [7]. Furthermore, Alvares et al. [46] found no benefit on muscular performance during resistance training following administration of 6 g of arginine. Aguiar et al. [47] also demonstrated no effect of acute arginine supplementation (8 g) on isometric peak torque production of the leg extensors. Acute citrulline supplementation at the recommended ergogenic level of 6–8 g has been shown to improve resistance training performance [48], but the data on isometric strength are limited. One study [49], however, demonstrated that 8 g of citrulline had no effect on isometric force for mid-thigh pull in resistance-trained subjects. Therefore, it is unlikely that the arginine or citrulline, at levels below the ergogenic threshold, contributed to the greater isometric strength demonstrated by the MIPS condition. Moreover, it is not uncommon for commercial MIPS products to contain insufficient amounts of key active ingredients [7]. The β-alanine content of 3.2 g in the MIPS in the current study was also provided at a dose below the suggested ergogenic level of 4–6 g [7]. In addition, β-alanine is recommended at these dosages for at least 2–4 weeks to improve exercise performance [13]. For example, chronic supplementation of β-alanine at ergogenic doses of 6.4 g·d^−1^ for four weeks has been shown to improve isometric endurance of the leg extensors [50]. To our knowledge, however, no studies have examined acute β-alanine supplementation on muscular performance due to these well-established chronic loading requirements [13].

The current MIPS product (121 ± 34 N·m) resulted in improved concentric peak torque production compared to the placebo (103 ± 27 N·m) during the PRE and POST fatigue isokinetic muscle actions at 60°·s^−1^, but no change for eccentric peak torque (124 ± 49 N·m vs. 120 ± 42 N·m, respectively). Thus, the ergogenic effects of the current MIPS during shortening muscle actions of the quadriceps were independent of the fatigue state, whereas no benefit was observed with muscle lengthening contractions. There are limited data on the effectiveness of acute MIPS supplementation on isokinetic muscle actions. Kaczka et al. [51], however, reported significantly greater concentric, isokinetic peak torque values and total work completed (across five repetitions) for both leg extension and flexion at 60°·s^−1^ following MIPS ingestion (L-citrulline, 3 g; β-alanine, 2 g; taurine, 750 mg; L-arginine, 500 mg; L-tyrosine, 500 mg; caffeine, 300 mg; guarana extract, 200 mg; barley-derived hordenine extract, 150 mg; capsaicin extract, 25 mg; black pepper extract, 7.5 mg; Huperzia serrata extract, 3 mg) compared to placebo. In contrast, Bergstrom et al. [19] found no improvement in concentric, isokinetic peak torque for leg extension and flexion at 30°·s^−1^ following a fatigue protocol (two, 3-min all-out critical power cycle ergometer tests and four supersets of lower body resistance training) during the MIPS condition (L-citrulline DL-malate, 6 g; L-leucine, 4 g; D-aspartic acid, 3 g; creatine hydrochloride, 2 g; β-alanine, 1.6 g; L-tyrosine, 1.2 g; agmatine sulfate, 500 mg; caffeine, 350 mg; phosphatidylserine, 125 mg; bioperine black pepper extract, 5 mg; huperzine serrata extract, 100 mcg; other vitamins and minerals) versus the placebo condition. Furthermore, no acute effects of MIPS supplementation were reported [18] during the concentric and eccentric phases of a maximal isokinetic squat with caffeinated and identical non-caffeinated products. Tinsley et al. [20] also found no differences in maximal concentric or eccentric torque production during slow (4-sec concentric, 4-sec eccentric phases), isokinetic squats following the acute administration of caffeinated (citrulline malate, 6 g; creatine, 3 g; betaine, 2.5 g; alpha-glyceryl phosphoryl choline, 300 mg; huperzine A, 200 mcg; caffeine, 300 mg; β-alanine, 2 g; taurine, 1 g; N-acetyl L-cystine, 600 mg; *Beta vulgaris*, 500 mg; BCAAs, 4.5 g; bioperine, 5 mg) and non-caffeinated MIPS conditions. Collectively, the present findings and others [18,19,20,51] suggested that caffeine-based MIPS may enhance concentric, isokinetic peak torque of the lower body at 60°·s^−1^, but not at 30°·s^−1^ or slower. It is possible that the ergogenic effect of MIPS products at ≥60°·s^−1^, compared to 30°·s^−1^, can be attributed to the increased neural and metabolic demands of contraction as movement velocity increases. Specifically, concentric torque performance becomes more dependent on greater motor unit recruitment, excitation-contraction coupling, and phosphagen resynthesis with faster contractions [52]. These factors would theoretically be enhanced by caffeine, β-alanine, and arginine/citrulline through enhanced central drive, buffering capacity, and blood flow. During slower velocity contractions (≤30°·s^−1^), however, concentric torque is mainly limited by cross-bridge mechanics [52] and may subsequently be less susceptible to ergogenic enhancement.

It is also possible that preferential improvement in concentric, but not eccentric, isokinetic peak torque relates to the distinct metabolic and neural demands of these different contraction types. For example, concentric muscle actions rely more heavily on ATP turnover and central motor drive, both of which may be enhanced by caffeine (as well as citrulline, arginine, and β-alanine with proper dosing) through increased excitability, energy availability, and buffering capacity [11,13,41,53,54]. In contrast, eccentric muscle actions are more mechanically efficient, requiring less metabolic support and greater reliance on passive elasticity [33]. Moreover, neural inhibitory mechanisms that limit maximal activation during eccentric muscle actions [33] may not be overcome by acute supplementation.

Our findings should be interpreted with caution due to some limitations. First, the MIPS was administered 30 min prior to exercise in accordance with the manufacturer’s recommendations. However, caffeine, the primary active ingredient, typically reaches peak plasma concentrations approximately 30–60 min post-ingestion [11], which likely occurred after the peak torque assessments. Second, the findings may not be generalizable to females, as only male participants were included. In addition, only one familiarization session was provided for the subjects to practice exerting maximal effort during isometric, concentric, and eccentric muscle actions using the isokinetic dynamometer. Thus, it is possible that additional orientation sessions would have resulted in greater peak torque values. Finally, the isokinetic velocity (60°·s^−1^) utilized in the fatigue protocol may not accurately represent the movement speeds commonly observed in athletic settings. Future studies should examine the acute effects of MIPS on concentric and eccentric peak torque across a wide range of isokinetic velocities, including those more closely associated with sports performance. Furthermore, the isolated effects of individual ingredients common to MIPS formulations on peak torque and muscle excitation should be assessed to provide better insight into the ergogenic properties of these products.

In conclusion, the present findings demonstrated that an acute dose of the MIPS enhanced isometric and concentric but not eccentric peak torque of the leg extensors before and after the isokinetic protocol, indicating improved force production under both rested and fatigued conditions. These ergogenic effects on peak torque were not reflected by changes in the amplitude or frequency contents of the EMG signal from the vastus lateralis, rectus femoris, or vastus medialis between the MIPS and placebo conditions. For practical application, administration of the current MIPS product 30 min prior to training or competition may increase performance in strength and power-focused movements involving isometric or concentric muscle actions. Trainers and coaches should monitor the frequency of MIPS utilization to prevent habituation and preserve the acute ergogenic response.

## Figures and Tables

**Table 1:** Normalized electromyographic amplitude values (mean ± SD) for the multi-ingredient preworkout supplement (MIPS) and placebo conditions.

		MIPS		Placebo	
	
		PRE	POST	PRE	POST
	
					
Isometric					
Vastus medialis (%)		100.0 ± 0.0	143.3 ± 71.9 *	100.0 ± 0.0	146.4 ± 79.5 *
Rectus femoris (%)		100.0 ± 0.0	157.1 ± 62.7 *	100.0 ± 0.0	141.0 ± 67.2 *
Vastus lateralis (%)		100.0 ± 0.0	149.8 ± 60.9 *	100.0 ± 0.0	136.9 ± 75.7 *
Concentric					
Vastus medialis (%)		123.7 ± 84.2	135.1 ± 90.2	112.9 ± 32.1	160.9 ± 141.6
Rectus femoris (%)		105.3 ± 37.3	133.4 ± 64.6	103.7 ± 28.3	126.3 ± 69.1
Vastus lateralis (%)		92.4 ± 29.9	120.3 ± 50.9	96.0 ± 35.0	112.5 ± 63.2
Eccentric					
Vastus medialis (%)		147.8 ± 112.2	156.5 ± 134.1	104.0 ± 32.4	144.9 ± 83.0
Rectus femoris (%)		100.0 ± 26.9	121.1 ± 58.3	96.6 ± 30.4	121.1 ± 62.0
Vastus lateralis (%)		89.9 ± 24.5	101.9 ± 45.3	92.5 ± 42.5	113.1 ± 57.6
					

All values expressed as relative to 100% of PRE value of isometric maximum voluntary contraction. * Significant (*p* < 0.05) main effect for time (PRE < POST).

**Table 2:** Normalized electromyographic median power frequency values (mean ± SD) for the multi-ingredient preworkout supplement (MIPS) and placebo conditions.

		MIPS		Placebo	
	
		PRE	POST	PRE	POST
	
					
Isometric					
Vastus medialis (%)		100.0 ± 0.0	95.5 ± 23.0	100.0 ± 0.0	114.1 ± 25.8
Rectus femoris (%)		100.0 ± 0.0	94.6 ± 32.3	100.0 ± 0.0	112.5 ± 38.6
Vastus lateralis (%)		100.0 ± 0.0	95.0 ± 22.8	100.0 ± 0.0	108.2 ± 11.0
Concentric					
Vastus medialis (%)		102.1 ± 12.2	95.6 ± 26.1 *	108.6 ± 20.1	101.0 ± 18.4 *
Rectus femoris (%)		95.3 ± 19.9	83.1 ± 20.1 *	113.8 ± 29.9	98.1 ± 36.1 *
Vastus lateralis (%)		99.4 ± 13.4	93.2 ± 12.7	110.5 ± 17.5	105.8 ± 13.1
Eccentric					
Vastus medialis (%)		97.1 ± 21.9	90.2 ± 18.3	105.1 ± 21.5	97.2 ± 17.8
Rectus femoris (%)		93.9 ± 25.0	82.8 ± 19.4	106.5 ± 24.4	97.7 ± 45.0
Vastus lateralis (%)		98.7 ± 14.1	94.6 ± 12.3	107.3 ± 21.4	105.3 ± 16.1
					

All values expressed as relative to 100% of PRE value of isometric maximum voluntary contraction. * Significant (*p* < 0.05) main effect for time (PRE > POST).

## Data Availability

The raw data supporting the conclusions of this article will be made available by the authors on request.

## Abbreviations

The following abbreviations are used in this manuscript:

MIPS	Multi-ingredient preworkout supplement
MVC	Maximum voluntary contraction
1-RM	One repetition maximum
EMG	Electromyography
MDF	Median power frequency
ANOVA	Analysis of variance
SD	Standard deviation
